# Comparison of three-dimensional heads-up system versus traditional microscopic system in medical education for vitreoretinal surgeries: a prospective study

**DOI:** 10.1186/s12909-024-05233-4

**Published:** 2024-03-15

**Authors:** Xin-yu Zhao, Qing Zhao, Ning-ning Li, Chu-ting Wang, Yin-han Wang, Li-hui Meng, Han-yi Min, You-xin Chen

**Affiliations:** 1 Department of Ophthalmology, Peking Union Medical College Hospital, Chinese Academy of Medical Sciences, No.1 Shuaifuyuan, Wangfujing, Dongcheng District, 100730 Beijing, China; 2 Key Laboratory of Ocular Fundus Diseases, Chinese Academy of Medical Sciences & Peking Union Medical College, Beijing, China; 3 Department of Operating Room, Peking Union Medical College Hospital, Chinese Academy of Medical Sciences, Beijing, China

**Keywords:** Three-dimensional heads-up surgery, Traditional microscopic system, Vitreoretinal surgery, Medical education

## Abstract

**Background:**

To compare the value and efficiency of the three-dimensional (3D) heads-up surgical system and traditional microscopic (TM) system in teaching and learning vitreoretinal surgeries.

**Methods:**

Twenty ophthalmologists and scrub nurses were recruited as teachers, and 45 junior ophthalmology residents and trainee doctors, trainee nurses, and medical students were recruited as observers. Each teacher and observer were assigned to both a 3D-assisted and TM-assisted vitreoretinal surgery and then asked to complete satisfaction questionnaires for both surgical systems at the end of each surgery.

**Results:**

The 3D heads-up surgical system was rated significantly higher in most of the subscales and overall satisfaction score by both teachers and observers (*P* < 0.05). However, ratings for instrument adjustment were significantly higher in the TM group compared to the 3D group for junior ophthalmology residents and trainee doctors (6.1 ± 1.7 vs. 8.8 ± 1.1, *P* < 0.001).

**Conclusions:**

The 3D heads-up surgical system has great didactical value in the medical education of vitreoretinal surgeries, but it is important to consider the specific needs of different learners when choosing between the two systems.

**Trial registration:**

Not applicable.

**Supplementary Information:**

The online version contains supplementary material available at 10.1186/s12909-024-05233-4.

## Background

Since its first application in vitreoretinal surgeries in 2016, [1] the three-dimensional (3D) heads-up surgical system has gained increasing popularity in the treatment of vitreoretinal diseases, such as epiretinal membrane (ERM) and macular hole (MH). The traditional microscopic (TM) system needs prolonged static unnatural neck-bent positions of the ophthalmologists and might cause severe musculoskeletal discomfort [2]. Instead, by using the 3D heads-up system and wearing polarized 3D glasses, surgeons could turn their heads up and view the surgical field on the 3D monitor without looking through microscope eyepieces in the neck-bent position [3]. The 3D heads-up system has reduced endo-illumination and the consequent retinal phototoxicity, extended depth of field, enhanced stereoscopic effect, and smoother surgical team communication [4–6]. Recently, further studies have reported that 3D heads-up surgeries have comparable efficacy and safety to the TM surgeries [7–12]. Furthermore, the current 3D technology has a latency of less than 70ms, which is unlikely to jeopardize the surgical performance and usability [13]. 

Recent studies have gradually noticed the great teaching potentiality of the 3D heads-up surgical system in medical education, [14, 15] but a number of issues remain unresolved. Notably, the current evaluations of the educational value of the 3D system lacked specificity and only used general statements in questionnaires, such as “educational value”, “teaching potential”, “teaching”, or “satisfaction” [11, 14]. Therefore, more detailed and specific evaluations were warranted. For the clinical teaching surgeons and nurses, some indices like interest and enthusiasm in teaching, teaching atmosphere and interaction, deep and scientific thinking, and others were very critical in the teaching process. Additionally, current reports on 3D heads-up surgeries mainly focus on the feedback from surgeons and medical interns, while the feelings and experiences of nurses and other observers are largely overlooked. Their understanding of the anatomical structure, surgical procedures and surgical cooperation, active operation cooperation, and surgical instrument recognition was also very vital for better teaching efficiency. Furthermore, no study exists to evaluate the teaching potentiality of the 3D system in the more complicated vitreoretinal surgeries, of which the training process usually takes several years. A more comprehensive assessment would enhance our understanding of the educational value of the 3D heads-up surgical system in vitreoretinal surgeries, given these deficiencies.

Accordingly, we applied the questionnaires customized according to the role of the respondents, for a better and comprehensive evaluation of the 3D heads-up surgical system in teaching and learning vitreoretinal surgeries.

## Materials and methods

### Study design

This prospective comparative study was conducted in accordance with the Declaration of Helsinki and approved by the Institutional Review Board of Peking Union Medical College Hospital (PUMCH) (approval number: K4849). Written informed consent for the agreement on the detailed operation and the instruments used in the surgery was obtained from all recruited patients.

### Sample size calculation

Based on our preliminary questionnaire evaluation, the mean general satisfaction scores among clinical teaching surgeons were 8.0 in the 3D group and 6.0 in the TM group. We set a Type I error level of 0.05 and a power level of 0.90 for our analysis. Under these assumptions, the minimum required sample size was determined to be 10 [16].

### Participant recruitment

Ten experienced ophthalmologists (as clinical teaching surgeons) and 10 scrub nurses (as clinical teaching nurses) were recruited from November 2022 to February 2023 from the Ophthalmology Department and Operating Room of PUMCH, respectively, forming the teacher group. The surgeons comprising the group of clinical teaching surgeons had a mean experience in vitreoretinal surgeries of 19.4 ± 6.3 years, with a range spanning from 10 to 32 years. Additionally, junior ophthalmology residents and trainee doctors (*N* = 15), trainee nurses (*N* = 15), and medical students (*N* = 15) were randomly recruited during the same time as the observer group. The recruited medical students had no prior basic knowledge or clinical work experience in ophthalmology and were considered as visitors. Each teacher and observer were assigned to both a 3D heads-up system-assisted vitreoretinal surgery and a TM system-assisted surgery.

### Surgical techniques

**Fig. 1:**
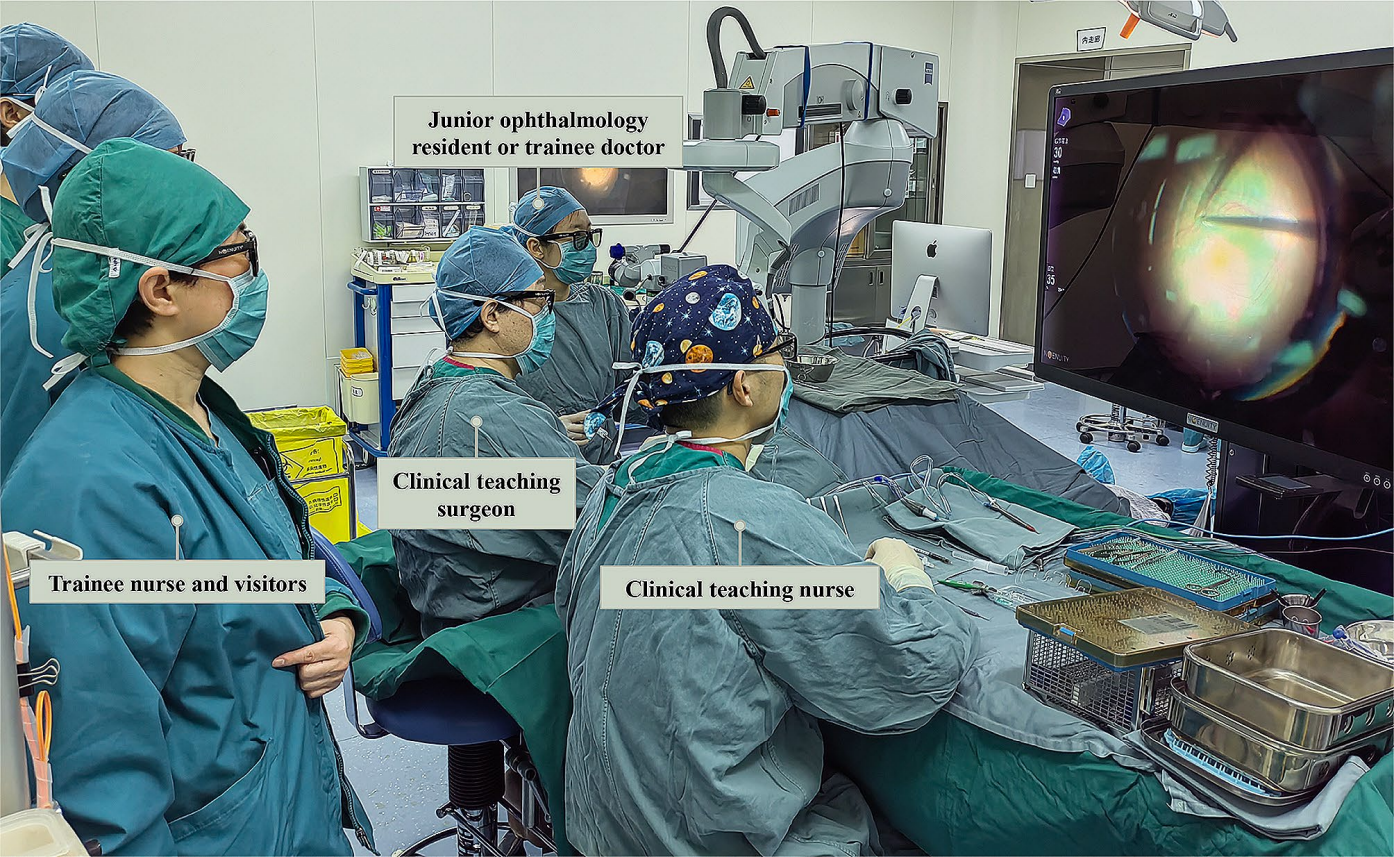
Teachers and observers wearing polarized 3D glasses in a surgery using 3D heads-up surgical system

Only vitreoretinal surgeries were assigned to teachers and observers, including surgeries for ERM, vitreomacular traction syndrome, VH, tractional and rhegmatogenous retinal detachments, macular hole, retinal detachment, pathologic myopic foveoschisis, silicone oil removal, and vitreous opacities. All surgeries were performed with the Alcon Constellation surgery system (Alcon Laboratories, Inc. Fort Worth, TX, USA). The TM group used the traditional microscopic system (OPMI-Lumera 700 with ReSight, Carl Zeiss Meditec AG, Jena, Germany), and the 3D group used the Alcon NGENUITY® 3D Visualization System (Alcon Laboratories, Inc. Fort Worth, TX, USA) (see Fig. 1). All patients underwent standard 23-gauge, 25-gauge or 27-gauge three-port pars plana vitrectomy (PPV) under local retrobulbar anesthesia or general anesthesia, with detailed surgical procedures varying depending on the surgical indications.

### Questionnaire evaluation

Questionnaires were designed to evaluate the satisfaction of all participants, respectively (see Supplementary material1-5). The reliability and validity of our self-designed questionnaires were evaluated using Cronbach’s alpha, where a value of 0.70 or higher indicates acceptable reliability, and the Kaiser-Meyer-Olkin test, where a value closer to 1 indicates stronger validity. The obtained values of 0.827 and 0.836 respectively indicated both acceptable reliability and validity. At the end of each surgery, each participant completed the questionnaire and rated their satisfaction on a continuous scale of 1 to 10, representing low to excellent, for both types of surgery. The clinical teaching surgeons and nurses rated parameters about their teaching experience and comfort, as well as the educational value of 3D and TM surgeries. Junior ophthalmology residents and trainee doctors, trainee nurses, and visitors rated parameters about their learning experience on observing surgeries using these two techniques, with detailed parameters varying depending on their role. An exam was scheduled for ophthalmology residents and trainee doctors, as well as trainee nurses, to objectively evaluate their understanding and knowledge about the fundus anatomy, features of the diseases and related surgical details, and their exam scores were recorded.   d 

**Table 1:** Comparison of teacher’s questionnaire scores between TM and 3D groups [This table should be placed below the first paragraph of the Results section]

		TM	3D	P
**Clinical teaching surgeons (** ***N*** ** = 10)**				
	Interest and enthusiasm in teaching	5.1 ± 1.8	7.6 ± 1.5	0.003^*^
	Teaching atmosphere and interaction	4.6 ± 1.6	7.9 ± 1.3	< 0.001^*^
	Smooth communication	4.5 ± 1.5	8.1 ± 1.8	< 0.001^*^
	Positive feedback	5.3 ± 1.7	8.2 ± 1.5	< 0.001^*^
	Deep and scienctific thinking	5.6 ± 1.9	8.0 ± 1.2	0.003^*^
	Expanding teaching contents	4.1 ± 1.5	8.5 ± 1.4	< 0.001^*^
	The explanation of anatomical structure	5.8 ± 1.6	8.6 ± 1.1	< 0.001^*^
	The explanation of surgical procedures	6.2 ± 1.3	8.2 ± 1.3	0.003^*^
	The explanation of surgical cooperation	6.5 ± 1.5	7.2 ± 1.5	0.311
	The quality of surgical presentation	5.8 ± 0.9	8.0 ± 1.2	< 0.001^*^
	Satisfaction with surgical cooperation	7.7 ± 1.2	7.3 ± 1.4	0.502
	Comfort level of teaching	6.0 ± 1.6	8.8 ± 0.9	< 0.001^*^
	General satisfaction	5.5 ± 1.2	8.5 ± 0.9	< 0.001^*^
	**Overall score**	**72.7 ± 19.3**	**104.9 ± 17.0**	**< 0.001** ^*^
**Clinical teaching nurses (** ***N*** ** = 10)**				
	Interest and enthusiasm in teaching	5.8 ± 1.4	9.0 ± 0.8	< 0.001^*^
	Teaching atmosphere and interaction	4.8 ± 1.2	8.4 ± 1.1	< 0.001^*^
	Smooth communication	4.4 ± 1.8	8.2 ± 0.9	< 0.001^*^
	Positive feedback	6.5 ± 1.5	8.5 ± 1.4	0.006^*^
	Deep and scienctific thinking	5.5 ± 1.4	8.6 ± 1.2	< 0.001^*^
	Expanding teaching contents	4.8 ± 1.9	7.8 ± 1.9	0.002^*^
	The explanation of surgical procedures	6.5 ± 2.0	8.5 ± 1.2	0.014^*^
	The explanation of surgical cooperation	6.2 ± 1.0	8.4 ± 1.4	< 0.001^*^
	The interpretation of surgical instrument	7.0 ± 1.5	7.2 ± 1.7	0.784
	The quality of surgical and cooperative presentation	6.6 ± 1.4	8.2 ± 1.3	0.016^*^
	Comfort level of teaching	5.5 ± 2.0	8.0 ± 1.1	0.003^*^
	General satisfaction	5.0 ± 2.1	8.5 ± 1.2	< 0.001^*^
	**Overall score**	**68.6 ± 19.2**	**99.3 ± 15.2**	**< 0.001** ^*^

*P < 0.05

*Abbreviations* TM, traditional microscopic; 3D, three-dimensional

**Table 2:** Comparison of observer’s questionnaire scores between TM and 3D groups [This table should be placed below the second paragraph of the Results section]

		TM	3D	P
**Junior ophthalmology residents and trainee doctors (** ***N*** ** = 15)**				
	Interest and enthusiasm in learning	5.2 ± 1.3	8.7 ± 1.1	< 0.001^*^
	Learning atmosphere and interaction	5.5 ± 1.5	8.9 ± 0.9	< 0.001^*^
	Deep and scientific thinking	5.9 ± 1.6	8.1 ± 1.0	< 0.001^*^
	The understanding of anatomical structure	5.4 ± 1.4	7.6 ± 0.9	< 0.001^*^
	The understanding of surgical procedures	6.6 ± 1.4	7.4 ± 1.3	0.116
	The understanding of surgical cooperation	6.2 ± 1.8	7.7 ± 1.1	0.010^*^
	Active operation cooperation	7.9 ± 1.6	7.6 ± 1.4	0.589
	Surgical Instrument recognition	8.1 ± 0.9	8.2 ± 1.2	0.798
	Instrument adjustment	8.8 ± 1.1	6.1 ± 1.7	< 0.001^*^
	Resolution of the surgical field	6.2 ± 1.8	8.7 ± 1.1	< 0.001^*^
	Stereoscopic sensation	8.5 ± 1.6	8.3 ± 1.4	0.718
	Magnification	6.1 ± 1.6	7.9 ± 1.3	0.002^*^
	Depth of Field	8.3 ± 1.4	8.1 ± 1.6	0.718
	Visual field	7.6 ± 1.3	7.3 ± 1.2	0.527
	Time latency between surgical interventions and their visualization	8.5 ± 1.3	8.4 ± 1.1	0.822
	Comfort level of learning	6.4 ± 1.8	8.8 ± 0.9	< 0.001^*^
	General satisfaction	7.1 ± 1.2	8.3 ± 1.0	0.006^*^
	The confidence in mastery of surgical procedures	5.5 ± 1.5	6.9 ± 1.3	0.011^*^
	Exam Score	7.1 ± 1.3	7.4 ± 1.2	0.517
	**Overall score**	**130.9 ± 27.4**	**150.4 ± 22.7**	**0.043** ^*^
**Trainee nurses (** ***N*** ** = 15)**				
	Interest and enthusiasm in learning	5.4 ± 2.0	7.1 ± 1.5	0.014^*^
	Learning atmosphere and interaction	6.1 ± 1.1	7.7 ± 1.2	< 0.001^*^
	Deep and scientific thinking	5.8 ± 1.3	8.2 ± 1.7	< 0.001^*^
	The understanding of anatomical structure	4.4 ± 2.0	6.7 ± 1.8	0.003^*^
	The understanding of surgical procedures	5.7 ± 1.3	7.2 ± 1.4	0.005^*^
	The understanding of surgical cooperation	6.6 ± 1.0	8.4 ± 0.9	< 0.001^*^
	Surgical Instrument recognition	7.2 ± 1.2	8.5 ± 0.9	0.002^*^
	Instrument preparation	7.7 ± 1.4	7.8 ± 1.6	0.857
	Active operation cooperation	7.4 ± 1.6	7.7 ± 1.5	0.600
	The quality of surgical observation	4.2 ± 2.1	8.5 ± 0.8	< 0.001^*^
	Comfort level of learning	5.6 ± 1.6	8.7 ± 0.8	< 0.001^*^
	Visual field	5.1 ± 1.6	8.2 ± 1.1	< 0.001^*^
	Stereoscopic sensation	3.9 ± 1.8	8.3 ± 1.0	< 0.001^*^
	General satisfaction	5.1 ± 2.0	8.2 ± 1.3	< 0.001^*^
	The confidence in mastery of surgical procedures	6.2 ± 1.9	7.9 ± 1.4	0.009^*^
	Exam Score	5.3 ± 1.3	7.7 ± 1.5	< 0.001^*^
	**Overall score**	**91.7 ± 25.2**	**126.8 ± 20.4**	**< 0.001** ^*^
**Visitors (** ***N*** ** = 15)**				
	Interest and enthusiasm in observation	3.9 ± 2.1	8.1 ± 1.1	< 0.001^*^
	Learning atmosphere and interaction	4.2 ± 1.3	7.8 ± 1.8	< 0.001^*^
	Deep and scientific thinking	3.4 ± 1.5	8.0 ± 1.3	< 0.001^*^
	The understanding of anatomical structure	3.6 ± 1.0	7.6 ± 1.7	< 0.001^*^
	The understanding of surgical procedures	4.1 ± 1.4	7.7 ± 1.5	< 0.001^*^
	The quality of surgical observation	4.2 ± 1.2	8.1 ± 1.1	< 0.001^*^
	Visual field	4.4 ± 1.3	7.0 ± 1.8	< 0.001^*^
	Stereoscopic sensation	3.1 ± 1.1	8.2 ± 1.0	< 0.001^*^
	General satisfaction	4.0 ± 1.5	8.5 ± 1.3	< 0.001^*^
	**Overall score**	**34.9 ± 12.4**	**71.0 ± 12.6**	**< 0.001** ^*^

*P < 0.05

*Abbreviations* TM, traditional microscopic; 3D, three-dimensional

### Statistical analysis

Continuous variables were summarized as mean ± standard deviation (SD), and categorical data were presented as frequency (percentages). Paired t-tests were used to compare each subgroup of questionnaire scores between TM and 3D groups for both teachers and observers. All statistical analyses were performed with Stata SE 12.0 software (StataCorp, College Station, TX, USA). The two-tailed *P* values < 0.05 were considered statistically significant.

## Results

### Teacher’s questionnaire scores

**Fig. 2:**
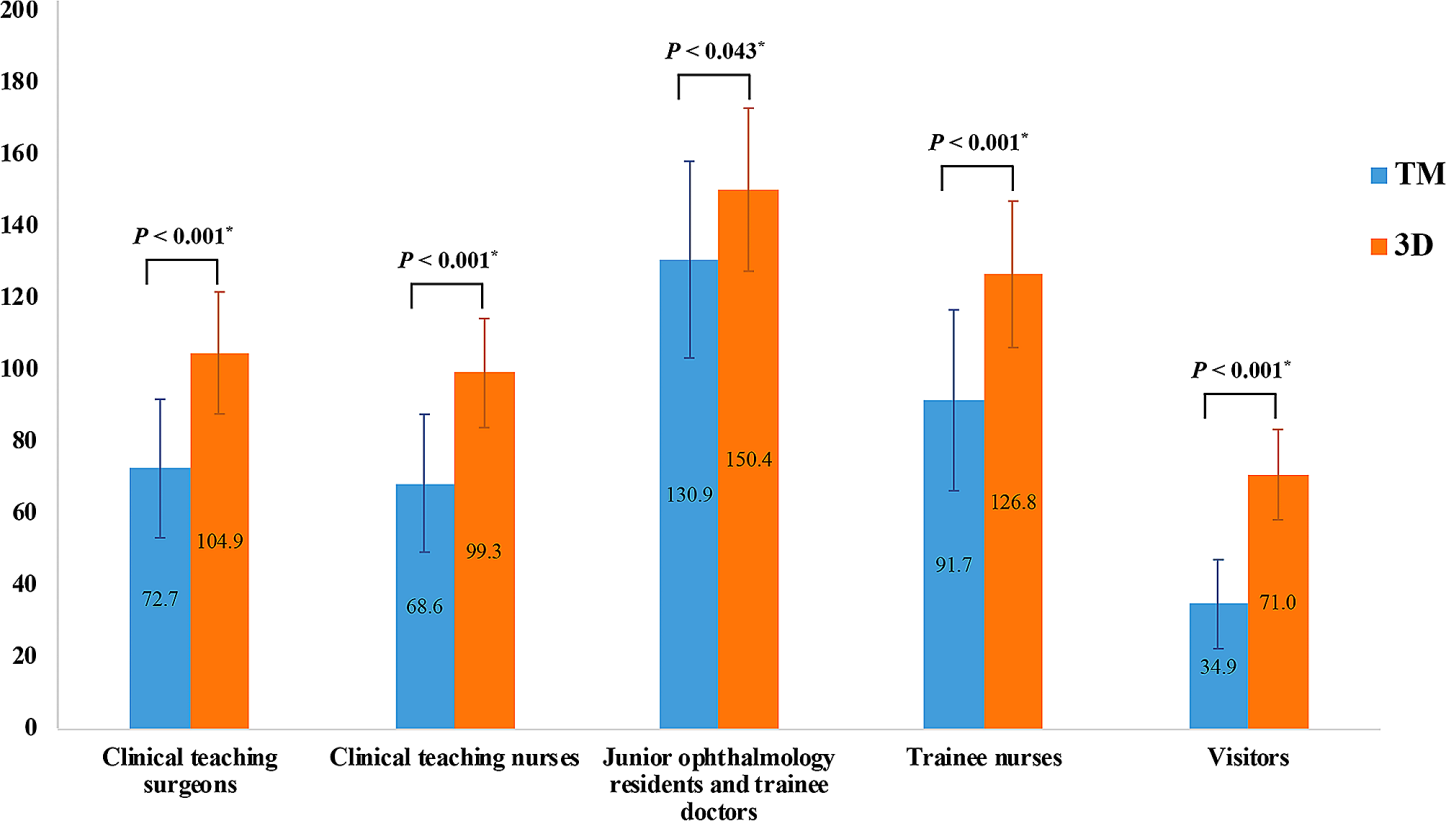
The overall satisfaction scores of teachers and observers to the TM and 3D groups

The detailed responses of the teachers to the questionnaire were shown in Table 1. The satisfaction score rated by the clinical teaching surgeons was significantly higher in the 3D group than in the TM group for all parameters (*P* < 0.05), except for the absence of a difference in the explanation of surgical cooperation (7.2 ± 1.5 vs. 6.5 ± 1.5, *P* = 0.311) and the satisfaction with surgical cooperation (7.3 ± 1.4 vs. 7.7 ± 1.2, *P* = 0.502). For clinical teaching nurses, no statistically significant difference was found regarding the interpretation of surgical instruments using these two techniques (7.2 ± 1.7 vs. 7.0 ± 1.5, *P* = 0.784), whereas 3D was significantly better than TM for the remaining items (*P* < 0.05). The overall satisfaction scores were higher for the 3D group compared to the TM group for both clinical teaching surgeons (104.9 ± 17.0 vs. 72.7 ± 19.3, *P* < 0.001) and nurses (99.3 ± 15.2 vs. 68.6 ± 19.2, *P* < 0.001) (see Fig. 2).

### Observer’s questionnaire scores

For junior ophthalmology residents and trainee doctors, the 3D group was rated better on most subscales (*P* < 0.05), except for worse instrument adjustment (6.1 ± 1.7 vs. 8.8 ± 1.1, *P* < 0.001). For trainee nurses, no statistically significant difference was found for instrument preparation (7.8 ± 1.6 vs. 7.7 ± 1.4, *P* = 0.857) and active operation cooperation (7.7 ± 1.5 vs. 7.4 ± 1.6, *P* = 0.600), but the 3D group was rated significantly better for the remaining items (*P* < 0.05). The visitors rated the 3D group significantly higher for each parameter in the satisfaction questionnaires (*P* < 0.05). The overall satisfaction scores were higher for the 3D group compared to the TM group for all groups of observers, including junior ophthalmology residents and trainee doctors (150.4 ± 22.7 vs. 130.9 ± 27.4, *P* = 0.043), trainee nurses (126.8 ± 20.4 vs. 91.7 ± 25.2, *P* < 0.001), and visitors (71.0 ± 12.6 vs. 34.9 ± 12.4, *P* < 0.001) (see Fig. 2). The detailed responses of the observers to the questionnaire are shown in Table 2.

## Discussion

In the present study, we compared the efficiency of the 3D heads-up surgical system and the TM system in the context of medical education for vitreoretinal surgeries. Based on the results, the 3D heads-up surgical system was rated significantly higher in most of the subscales and overall satisfaction score by both teachers and observers. However, when it came to instrument adjustment, the ratings were significantly higher in the TM group compared to the 3D group for junior ophthalmology residents and trainee doctors.

In TM-assisted surgeries, the surgeons could only introduce and explain the surgical related details based on 2D images on the monitor. Also, only the surgeons can have a high-grade stereo view of the surgical field, while the remaining observers could not appreciate the depth of field and the 3D view necessary for a better understanding of anatomical structure and surgical details. In contrast, the 3D heads-up surgical system allows all members of the surgical team to view the same live surgical image (see Fig. 1), which is especially helpful for observers who might not have access to a high-grade stereo view of the surgical field. Besides, its larger high-resolution screen provides a better 3D view of the anatomical structure and facilitated real-time instructions, which improves the teaching and learning experience. The present study found that the 3D heads-up surgical system was significantly superior to the TM system in terms of teaching/learning experience and comfort, including interest and enthusiasm in teaching/learning, teaching/learning atmosphere and interaction, deep and scientific thinking, the explanation/understanding of anatomical structure, comfort level of teaching/learning, and general satisfaction. Previous studies also reported the superior educational value of the 3D system over the TM system [17–19]. These findings suggested that the 3D heads-up surgical system could enhance observers’ understanding and knowledge retention of vitreoretinal surgeries by enabling them to view more surgical details with more comfortable experience.

The present study found no statistically significant difference between 3D and TM systems on the subscales of “surgical instrument recognition”, “instrument preparation”, and “the interpretation of surgical instrument” as rated by junior ophthalmology residents and trainee doctors, trainee nurses, and clinical teaching nurses, respectively. Similarly, Palácios et al. [20] reported similar technical feasibility between the two systems based on satisfaction questionnaires. Del Turco et al. [15] conducted a retrospective observational case series to investigate surgeon preferences for 3D and TM systems when performing retina, cataract, and corneal surgeries. Around 30% of surgeons found that the 3D surgical system was as simple as the TM system, which may be attributed to the similar complexity of surgical instruments used in both systems.

The controversy surrounding the comparison of system-related indexes between 3D and TM surgical systems has been ongoing for a while. The visual field, which refers to the maximum area visible at any given moment and is inversely proportional to magnification, has been a major point of contention. While some studies have reported a better field of view in the 3D heads-up system and higher depth of field even under high magnification regardless of the area focused, [1, 6, 18] others have reported no statistically significant difference in the visual field, depth of field, and image resolution between the 3D heads-up surgical system and the TM system [20]. The inconsistent findings in these studies may be attributed to various factors, such as the difference in surgical instrumentations, categories of surgeries, and the role of the respondents. The Alcon NGENUITY® 3D Visualization System was associated with significantly higher resolution of the surgical field, higher magnification, higher visual field, and better stereoscopic sensation than the TM system, according to observers in the present study, which is consistent with a previous study [7]. Interestingly, in an experimental study conducted by Eckardt et al. [1], most of the volunteers found that 3D images were sharper and with equal or higher resolution than TM images, despite the measurement of the resolution of TM with eyepieces being higher than the 3D system. This may be explained by the fact that all the light is sent to the camera in the 3D heads-up system by removing the eyepieces, which improves the quality of digital images.

It appears that the use of the 3D heads-up system during surgeries resulted in discomfort and lower scores in the subscale of “instrument adjustment” among junior ophthalmology residents and trainee doctors, who acted as the first assistants. The discomfort was caused by the need to uncomfortably rotate their heads to look at the screen and bear the inconsistent directions of the screen and instrument, which further increased the difficulties of performing the 3D heads-up surgery. Additionally, the uncomfortable ergonomics of the head position may have contributed to lower satisfaction scores of the 3D heads-up system in “operation cooperation” rated by junior ophthalmology residents and trainee doctors, although without statistical significance. Zhao et al. [7] and Rizzo et al. [14] have also reported dissatisfaction among the first assistants with the comfort or operation cooperation when evaluating the perceptions of the surgical team to the 3D heads-up surgical system. Recently, Bausch + Lomb, in collaboration with Heidelberg Engineering, introduced the innovative 3D heads-up surgical visualization platform, SeeLuma™. This platform features a C-shaped arm, enabling the screen to be positioned in front of the surgeon without obstruction from the camera. Additionally, this system supports multiple wireless displays, potentially offering the first assistant an additional screen. This feature may alleviate the discomfort experienced when using our current 3D heads-up system. Further improvements are needed in the near future to make the 3D system more comfortable for every member of the surgical team.

The high-resolution screen was placed approximately 2.2 m away from the surgeons when performing 3D heads-up surgeries. It appears that there are pros and cons to using the 3D heads-up surgical system versus TM system of looking down through eyepieces during surgery. One advantage of the heads-up system is that surgeons can more conveniently receive instruments from scrub nurses by observing from the corners of their eyes, which may improve operational efficiency. However, there is a disadvantage in that surgeons are more susceptible to being disturbed by the movements of other members in the operating room because they were not close enough to the 3D screen [21]. This can be particularly problematic during delicate surgical procedures. Therefore, it is important for surgeons to carefully consider the advantages and disadvantages of each system before deciding which to use for a given procedure.

Several limitations should indeed be considered when interpreting the findings. First, the sample size in this study was limited, which may not necessarily represent the broader population and might have limited generalizability. Furthermore, while the study provided valuable insights into the implementation of the 3D heads-up surgical system in the medical education of vitreoretinal surgeries, it did not investigate its efficiency in teaching and learning specific types of surgeries. Therefore, future researches are warranted to examine its value in teaching different surgical techniques and determine if the system is more effective for some surgeries than others.

## Conclusions

In summary, the 3D heads-up surgical system was superior to TM surgery in most subscales and overall scores in satisfaction questionnaires rated by teachers and observers. However, it showed inferiority in instrument adjustment rated by junior ophthalmology residents and trainee doctors. Overall, the study demonstrates that the 3D heads-up surgical system has great didactical value in the medical education of vitreoretinal surgeries, but it is important to consider the specific needs of different learners when choosing between the two systems.

### Electronic supplementary material

Below is the link to the electronic supplementary material.

Supplementary Material 1-5

## Data Availability

The datasets used and/or analysed during the current study are available from the corresponding author on reasonable request.

## Abbreviations

ERM: epiretinal membrane
MH: macular hole
PPV: pars plana vitrectomy
SD: standard deviation
TM: traditional microscopic
3D: three-dimensional

## References

[1] Eckardt C, Paulo EB, HEADS-UP SURGERY FOR VITREORETINAL (2016). PROCEDURES: an experimental and clinical study. Retina Jan.

[2] Diaconita V, Uhlman K, Mao A, Mather R (2019). Survey of occupational musculoskeletal pain and injury in Canadian ophthalmology. Can J Ophthalmol Jun.

[3] Zhang Z, Wang L, Wei Y, Fang D, Fan S, Zhang S (2019). The preliminary experiences with three-dimensional Heads-Up Display viewing system for vitreoretinal surgery under various status. Curr Eye Res Jan.

[4] Nariai Y, Horiguchi M, Mizuguchi T, Sakurai R, Tanikawa A (2021). Comparison of microscopic illumination between a three-dimensional heads-up system and eyepiece in cataract surgery. Eur J Ophthalmol Jul.

[5] Rosenberg ED, Nuzbrokh Y, Sippel KC (2021). Efficacy of 3D digital visualization in minimizing coaxial illumination and phototoxic potential in cataract surgery: pilot study. J Cataract Refract Surg Mar.

[6] Ohno H (2019). Utility of three-dimensional Heads-Up surgery in cataract and minimally invasive Glaucoma surgeries. Clin Ophthalmol.

[7] Zhao XY, Zhao Q, Li NN (2023). Surgery-related characteristics, efficacy, safety and surgical team satisfaction of three-dimensional heads-up system versus traditional microscopic equipment for various vitreoretinal diseases. Graefes Arch Clin Exp Ophthalmol Mar.

[8] Wang K, Song F, Zhang L (2021). Three-dimensional heads-up cataract surgery using Femtosecond laser: efficiency, efficacy, Safety, and Medical Education-A Randomized Clinical Trial. Transl Vis Sci Technol Aug.

[9] Piccirillo V, Sbordone S, Sorgente F (2021). To estimate the safety and efficacy of a 3-D visualization helmet for vitreoretinal surgery. Acta Ophthalmol May.

[10] Palácios RM, Maia A, Farah ME, Maia M (2019). Learning curve of three-dimensional heads-up vitreoretinal surgery for treating macular holes: a prospective study. Int Ophthalmol Oct.

[11] Romano MR, Cennamo G, Comune C (2018). Evaluation of 3D heads-up vitrectomy: outcomes of psychometric skills testing and surgeon satisfaction. Eye (Lond) Jun.

[12] Ripa M, Kopsacheilis N, Kanellopoulou K, Nomikarios M, Motta L. Three-Dimensional Heads-Up vs. Standard operating microscope for cataract surgery: a systematic review and Meta-analysis. Diagnostics (Basel) Aug. 2022;30(9). 10.3390/diagnostics12092100. 10.3390/diagnostics12092100 PMC9497825 36140501

[13] Ta Kim D, Chow D. The effect of latency on surgical performance and usability in a three-dimensional heads-up display visualization system for vitreoretinal surgery. Graefes Arch Clin Exp Ophthalmol. Sep 2021;3. 10.1007/s00417-021-05388-6. 10.1007/s00417-021-05388-6 34477929

[14] Rizzo S, Abbruzzese G, Savastano A (2018). 3D SURGICAL VIEWING SYSTEM IN OPHTHALMOLOGY: perceptions of the Surgical Team. Retina Apr.

[15] Del Turco C, D’Amico Ricci G, Dal Vecchio M (2021). Heads-up 3D eye surgery: safety outcomes and technological review after 2 years of day-to-day use. Eur J Ophthalmol Apr.

[16] Chow SCSJ, Wang H. Sample size calculation in Clinical Research. New York: Marcel Dekker. 2008.

[17] Coppola M, La Spina C, Rabiolo A, Querques G, Bandello F (2017). Heads-up 3D vision system for retinal detachment surgery. Int J Retina Vitreous.

[18] Mendez BM, Chiodo MV, Vandevender D, Patel PA (2016). Heads-up 3D Microscopy: an Ergonomic and Educational Approach to Microsurgery. Plast Reconstr Surg Glob Open May.

[19] Weinstock RJ, Diakonis VF, Schwartz AJ, Weinstock AJ (2019). Heads-up cataract surgery: Complication Rates, Surgical Duration, and comparison with traditional microscopes. J Refract Surg May.

[20] Palácios RM, Kayat KV, Morel C (2020). Clinical study on the initial experiences of French Vitreoretinal surgeons with heads-up surgery. Curr Eye Res Oct.

[21] Berquet F, Henry A, Barbe C (2020). Comparing Heads-Up versus binocular microscope visualization systems in anterior and posterior segment surgeries: a retrospective study. Ophthalmologica.

