# Supplementary material for: Comparison of three-dimensional heads-up system versus traditional microscopic system in medical education for vitreoretinal surgeries: a prospective study
Source: BMC Med Educ. 2024 Mar 15;24:290. doi: 10.1186/s12909-024-05233-4 (PMC10943918; doi:10.1186/s12909-024-05233-4)
Supplement: Supplementary file 1 — Supplementary Material 1-5 [file 12909_2024_5233_MOESM1_ESM.docx]

**Supplementary material 1. Satisfaction survey on the surgical system in medical education for vitreoretinal surgery (designed for clinical teaching surgeons)**

**Dear participants (clinical teaching surgeons):**

**Thank you for taking the time to participate in our survey. This questionnaire aims to assess your satisfaction with the surgical system in teaching vitreoretinal surgery. Your input is invaluable for us to improve our teaching methods and enhance the learning experience for future students.**

1. Your name: ________
2. Years of experience in performing vitreoretinal surgeries: ________ years
3. Based on your experience and satisfaction level when performing and teaching vitreoretinal surgeries using this surgical system, please rate the following aspects of the teaching session on a scale from 0 to 10, where 0 means very poor/strongly disagree and 10 means excellent/strongly agree:

3.1 Using this surgical system sparks my interest and enthusiasm in teaching:

0 1 2 3 4 5 6 7 8 9 10

3.2 Using this surgical system creates a positive teaching atmosphere and fosters good interaction with students:

0 1 2 3 4 5 6 7 8 9 10

3.3 Using this surgical system facilitates smooth and unobstructed communication between me and the students:

0 1 2 3 4 5 6 7 8 9 10

3.4 Using this surgical system helps me receive effective feedback from students:

0 1 2 3 4 5 6 7 8 9 10

3.5 Using this surgical system encourages students to engage in deep and scientific thinking [such as asking questions and thinking actively and critically]:

0 1 2 3 4 5 6 7 8 9 10

3.6 Using this surgical system makes it easy for me to expand on teaching content:

0 1 2 3 4 5 6 7 8 9 10

3.7 With this surgical system, I can easily demonstrate the anatomical structures to students in real-time:

0 1 2 3 4 5 6 7 8 9 10

3.8 With this surgical system, I can easily demonstrate the surgical procedures to students in real-time:

0 1 2 3 4 5 6 7 8 9 10

3.9 With this surgical system, I can easily demonstrate collaboration among surgeons, as well as between surgeons and other members of the surgical team to students:

0 1 2 3 4 5 6 7 8 9 10

3.10 I am satisfied with the surgical field displayed by this surgical system:

0 1 2 3 4 5 6 7 8 9 10

3.11 I am satisfied with the collaboration among surgeons as well as the collaboration between surgeons and other members of the surgical team using this surgical system:

0 1 2 3 4 5 6 7 8 9 10

3.12 I am satisfied with the teaching comfort when using this surgical system for vitreoretinal surgery:

0 1 2 3 4 5 6 7 8 9 10

3.13 I am generally satisfied with this surgical system:

0 1 2 3 4 5 6 7 8 9 10

**Please feel free to provide any additional comments or suggestions for improvement in the space provided below.**

**__________________________________________________________________________________**

**Thank you very much for your participation and valuable feedback.**

**Supplementary material 2. Satisfaction survey on the surgical system in medical education for vitreoretinal surgery (designed for clinical teaching nurses)**

**Dear participants (clinical teaching nurses):**

**Thank you for taking the time to participate in our survey. This questionnaire aims to assess your satisfaction with the surgical system in teaching how to assist with vitreoretinal surgery. Your input is invaluable for us to improve our teaching methods and enhance the learning experience for future students.**

1. Your name: ________
2. Your experience in assisting with vitreoretinal surgeries: ________ years
3. Based on your experience and satisfaction level when teaching how to assist with vitreoretinal surgeries using this surgical system, please rate the following aspects of the teaching session on a scale from 0 to 10, where 0 means very poor/strongly disagree and 10 means excellent/strongly agree:

3.1 Using this surgical system sparks my interest and enthusiasm in teaching:

0 1 2 3 4 5 6 7 8 9 10

3.2 Using this surgical system creates a positive teaching atmosphere and fosters good interaction with students:

0 1 2 3 4 5 6 7 8 9 10

3.3 Using this surgical system facilitates smooth and unobstructed communication between me and the students:

0 1 2 3 4 5 6 7 8 9 10

3.4 Using this surgical system helps me receive effective feedback from students:

0 1 2 3 4 5 6 7 8 9 10

3.5 Using this surgical system encourages students to engage in deep and scientific thinking [such as asking questions and thinking actively and critically]:

0 1 2 3 4 5 6 7 8 9 10

3.6 Using this surgical system makes it easy for me to expand on teaching content:

0 1 2 3 4 5 6 7 8 9 10

3.7 With this surgical system, I can easily demonstrate the surgical procedures to students in real-time:

0 1 2 3 4 5 6 7 8 9 10

3.8 With this surgical system, I can easily demonstrate collaboration between surgeons and instrument nurses to students:

0 1 2 3 4 5 6 7 8 9 10

3.9 With this surgical system, I can easily introduce the surgical system and all microsurgical instruments to my students:

0 1 2 3 4 5 6 7 8 9 10

3.10 I am satisfied with the quality of the presentation of the surgical procedures and the cooperation between surgeons and instrument nurses:

0 1 2 3 4 5 6 7 8 9 10

3.11 I am satisfied with the teaching comfort when using this surgical system for assisting vitreoretinal surgery:

0 1 2 3 4 5 6 7 8 9 10

3.12 I am generally satisfied with this surgical system:

0 1 2 3 4 5 6 7 8 9 10

**Please feel free to provide any additional comments or suggestions for improvement in the space provided below.**

**__________________________________________________________________________________**

**Thank you for your participation and valuable feedback.**

**Supplementary material 3. Satisfaction survey on the surgical system in medical education for vitreoretinal surgery (designed for junior ophthalmology residents and trainee doctors)**

**Dear participants (junior ophthalmology residents and trainee doctors):**

**Thank you for taking the time to participate in our survey. This questionnaire aims to assess your satisfaction with the surgical system in learning how to perform vitreoretinal surgery. Your input is invaluable for us to improve our teaching methods and enhance the learning experience for future students.**

1. Your name: ________
2. Your gender: ________
3. Based on your experience and satisfaction level when learning vitreoretinal surgeries using this surgical system, please rate the following aspects of the learning session on a scale from 0 to 10, where 0 means very poor/strongly disagree and 10 means excellent/strongly agree:

3.1 Using this surgical system fully ignites my interest and enthusiasm in learning:

0 1 2 3 4 5 6 7 8 9 10

3.2 Using this surgical system helps create a positive learning atmosphere and enables good interaction with the teacher:

0 1 2 3 4 5 6 7 8 9 10

3.3 Using this surgical system promotes deep and scientific thinking [such as asking questions and thinking actively and critically] for me:

0 1 2 3 4 5 6 7 8 9 10

3.4 With this surgical system, I can easily understand the anatomical structures being operated on:

0 1 2 3 4 5 6 7 8 9 10

3.5 With this surgical system, I can easily understand the surgical procedures being performed:

0 1 2 3 4 5 6 7 8 9 10

3.6 With this surgical system, I can easily understand the cooperation among surgeons:

0 1 2 3 4 5 6 7 8 9 10

3.7 With this surgical system, I can easily synchronize my thoughts with the primary surgeon and make surgical cooperation smoother:

0 1 2 3 4 5 6 7 8 9 10

3.8 With this surgical system, I can easily recognize microsurgical instruments accurately:

0 1 2 3 4 5 6 7 8 9 10

3.9 With this surgical system, I can easily adjust microsurgical instruments to optimal settings:

0 1 2 3 4 5 6 7 8 9 10

3.10 I am satisfied with the resolution of the surgical field displayed by this surgical system:

0 1 2 3 4 5 6 7 8 9 10

3.11 I am satisfied with the stereoscopic sensation displayed by this surgical system:

0 1 2 3 4 5 6 7 8 9 10

3.12 I am satisfied with the magnification displayed by this surgical system:

0 1 2 3 4 5 6 7 8 9 10

3.13 I am satisfied with the depth of field displayed by this surgical system:

0 1 2 3 4 5 6 7 8 9 10

3.14 I am satisfied with the surgical visual field displayed by this surgical system:

0 1 2 3 4 5 6 7 8 9 10

3.15 I believe the time latency between surgical interventions and their visualization of this surgical system is minimal:

0 1 2 3 4 5 6 7 8 9 10

3.16 I am satisfied with the comfort when learning vitreoretinal surgery using this surgical system:

0 1 2 3 4 5 6 7 8 9 10

3.17 I am generally satisfied with this surgical system:

0 1 2 3 4 5 6 7 8 9 10

3.18 I am confident in mastering the surgical procedures using this surgical system:

0 1 2 3 4 5 6 7 8 9 10

**Please feel free to provide any additional comments or suggestions for improvement in the space provided below.**

**__________________________________________________________________________________**

**Thank you for your participation and valuable feedback.**

**Supplementary material 4. Satisfaction survey on the surgical system in medical education for vitreoretinal surgery (designed for trainee nurses)**

**Dear participants (trainee nurses):**

**Thank you for taking the time to participate in our survey. This questionnaire aims to assess your satisfaction with the surgical system in learning how to assist with vitreoretinal surgery. Your input is invaluable for us to improve our teaching methods and enhance the learning experience for future students.**

1. Your name: ________
2. Your gender: ________
3. Based on your experience and satisfaction level when learning vitreoretinal surgeries using this surgical system, please rate the following aspects of the learning session on a scale from 0 to 10, where 0 means very poor/strongly disagree and 10 means excellent/strongly agree:

3.1 Using this surgical system fully ignites my interest and enthusiasm in learning:

0 1 2 3 4 5 6 7 8 9 10

3.2 Using this surgical system helps create a positive learning atmosphere and enables good interaction with the teacher:

0 1 2 3 4 5 6 7 8 9 10

3.3 Using this surgical system promotes deep and scientific thinking [such as asking questions and thinking actively and critically] for me:

0 1 2 3 4 5 6 7 8 9 10

3.4 With this surgical system, I can easily understand the anatomical structures being operated on:

0 1 2 3 4 5 6 7 8 9 10

3.5 With this surgical system, I can easily understand the surgical procedures being performed:

0 1 2 3 4 5 6 7 8 9 10

3.6 With this surgical system, I can easily understand the cooperation among surgeons, as well as between surgeons and instrument nurses:

0 1 2 3 4 5 6 7 8 9 10

3.7 With this surgical system, I can easily recognize microsurgical instruments accurately:

0 1 2 3 4 5 6 7 8 9 10

3.8 I can easily get the surgical system and the microsurgical instruments well prepared for surgeons:

0 1 2 3 4 5 6 7 8 9 10

3.9 With this surgical system, I can easily synchronize my thoughts with the surgeons and make surgical cooperation smoother:

0 1 2 3 4 5 6 7 8 9 10

3.10 I am satisfied with the quality of surgical observation displayed by this surgical system:

0 1 2 3 4 5 6 7 8 9 10

3.11 I am satisfied with the comfort when learning vitreoretinal surgery using this surgical system:

0 1 2 3 4 5 6 7 8 9 10

3.12 I am satisfied with the surgical visual field displayed by this surgical system:

0 1 2 3 4 5 6 7 8 9 10

3.13 I am satisfied with the stereoscopic sensation displayed by this surgical system:

0 1 2 3 4 5 6 7 8 9 10

3.14 I am generally satisfied with this surgical system:

0 1 2 3 4 5 6 7 8 9 10

3.15 I am confident in mastering the surgical procedures using this surgical system:

0 1 2 3 4 5 6 7 8 9 10

**Please feel free to provide any additional comments or suggestions for improvement in the space provided below.**

**__________________________________________________________________________________**

**Thank you for your participation and valuable feedback.**

**Supplementary material 5. Satisfaction survey on the surgical system in medical education for vitreoretinal surgery (designed for visitors)**

**Dear participants (visitors):**

**Thank you for taking the time to participate in our survey. This questionnaire aims to assess your satisfaction with the surgical system in observing vitreoretinal surgery. Your input is invaluable for us to improve our teaching methods and enhance the learning experience for future students.**

1. Your name: ________
2. Your gender: ________
3. Based on your experience and satisfaction level when observing vitreoretinal surgeries using this surgical system, please rate the following aspects of the observing session on a scale from 0 to 10, where 0 means very poor/strongly disagree and 10 means excellent/strongly agree:

3.1 Using this surgical system fully ignites my interest and enthusiasm in observing:

0 1 2 3 4 5 6 7 8 9 10

3.2 Using this surgical system helps create a positive learning atmosphere and enables good interaction between students and teachers:

0 1 2 3 4 5 6 7 8 9 10

3.3 Using this surgical system promotes deep and scientific thinking [such as asking questions and thinking actively and critically] for me:

0 1 2 3 4 5 6 7 8 9 10

3.4 With this surgical system, I can easily understand the anatomical structures being operated on:

0 1 2 3 4 5 6 7 8 9 10

3.5 With this surgical system, I can easily understand the surgical procedures being performed:

0 1 2 3 4 5 6 7 8 9 10

3.6 I am satisfied with the quality of surgical observation displayed by this surgical system:

0 1 2 3 4 5 6 7 8 9 10

3.7 I am satisfied with the surgical visual field displayed by this surgical system:

0 1 2 3 4 5 6 7 8 9 10

3.8 I am satisfied with the stereoscopic sensation displayed by this surgical system:

0 1 2 3 4 5 6 7 8 9 10

3.9 I am generally satisfied with this surgical system:

0 1 2 3 4 5 6 7 8 9 10

**Please feel free to provide any additional comments or suggestions for improvement in the space provided below.**

**__________________________________________________________________________________**

**Thank you for your participation and valuable feedback.**
